# Influence of childhood trauma and brain-derived neurotrophic factor Val66Met polymorphism on posttraumatic stress symptoms and cortical thickness

**DOI:** 10.1038/s41598-019-42563-6

**Published:** 2019-04-15

**Authors:** Min Jin Jin, Hyeonjin Jeon, Myoung Ho Hyun, Seung-Hwan Lee

**Affiliations:** 1 Clinical Emotion and Cognition Research Laboratory, Inje University, Goyang, Republic of Korea; 2 Department of Psychology, Chung-Ang University, Seoul, Republic of Korea; 3 Department of Psychiatry, Inje University, Ilsan-Paik Hospital, Goyang, Republic of Korea

## Abstract

Interaction between childhood trauma and genetic factors influences the pathophysiology of posttraumatic stress disorder (PTSD). This study examined the interaction effect of childhood trauma and brain-derived neurotrophic factor (BDNF) Val66Met polymorphism on PTSD symptoms and brain cortical thickness. A total of 216 participants (133 healthy volunteers and 83 PTSD patients) were recruited. T1-weighted structural magnetic resonance imaging, BDNF rs6265 genotyping through blood sampling, and clinical assessments including the childhood trauma questionnaire (CTQ) and posttraumatic stress disorder Checklist (PCL) were performed. A moderated regression analysis, two-way multivariate analysis of covariance, and correlation analysis were conducted. An interaction between the CTQ and the BDNF polymorphism significantly influenced PTSD symptom severity. In fact, people with rs6265 Val/Val genotype and higher CTQ scores showed higher PCL scores. Additionally, this interaction was significant on both left fusiform and transverse temporal gyri thickness. Furthermore, the thickness of both brain regions was significantly correlated with psychological symptoms including depression, anxiety, rumination, and cognitive emotion regulation methods; yet this was mainly observed in people with the Val/Val genotype. The interaction between childhood trauma and BDNF polymorphism significantly influences both PTSD symptoms and cortical thickness and the Val/Val genotype may increase the risk in Korean population.

## Introduction

Posttraumatic stress disorder (PTSD), a highly debilitating condition with severe symptoms, is influenced by both environmental and genetic factors^1^. Among many traumatic experiences, people who faced childhood trauma are more likely to develop PTSD symptoms^2–4^. Furthermore, childhood trauma is identified as an environmental factor which could contribute to PTSD symptoms when a genetic predisposition to childhood trauma is present, commonly referred to a gene-environment interaction^5^. The brain-derived neurotrophic factor (BDNF) is known to be one of the key candidate genes for PTSD^6–10^.

BDNF and its receptors play important roles in the regulation, differentiation, and maintenance of both the peripheral and central nervous systems^11^. The BDNF gene contains a functional single-nucleotide polymorphism (rs6265) that is associated with regulation of both transportation and secretion of BDNF in neurons^12^. Additionally, BDNF is renowned for its role in neuronal survival and growth-promoting actions in the central nervous system^13^. Specifically, the rs6265 polymorphism results in a valine (Val) to methionine (Met) substitution at codon 66 (Val66Met)^14^. Interestingly, the rs6265 was hypothesized to play an important role in fear learning^15^.

Although increasing evidence indicates that the rs6265 is involved in various psychiatric disorders including PTSD^16–19^, the results are inconsistent. In fact, several studies suggested that the Met allele may be related to increased PTSD susceptibility^8,10,20–22^, while the Val/Val genotype may have a protective role towards PTSD development^16^. However, other studies did not report any significant correlation between the rs6265 polymorphism and PTSD^23–26^. Further studies even suggested the Met allele to have a protective function and participants with the Val/Val genotype to show symptoms such as anxiety^27^, neuroticism^28^, obsessive-compulsive disorder^29–32^, and depression^27,33^.

Furthermore, up to date, the findings related to the interaction effect of BDNF rs6265 polymorphism and childhood traumatic experiences on brain structure are also contradictory. In fact, previous studies found that Met allele carriers to present thinner and smaller brain cortical structures. Additionally, Met carriers with a history of childhood adversity had significantly less gray matter in their subgenual anterior cingulate cortices^34^, smaller amygdala volumes, and less gray matter^35,36^ when compared to both Met carriers without any childhood adversity and Val/Val homozygotes with childhood adversity. However, other studies found the Val/Val genotype to be related to thinner and smaller brain cortical structures; maltreated Val/Val participants showed thinner rostral anterior cingulate cortices than non-maltreated Val/Val participants^37^, and Val/Val children with a history of traumatic experiences presented decreased hippocampal volume when compared to the Met allele children^38^. Some researchers claimed the reason behind such mixed to be racial differences^26^ and the relative rarity of the Met allele^19^. Only one Korean study reporting the interaction between BDNF and childhood trauma to have an effect on the severity of anxiety symptoms in healthy participants exists^39^. However, a study focusing on the interaction effect of BDNF and childhood trauma on cortical structures in Korean participants is yet to be conducted. Moreover, there are no studies on discovering the relationship between brain regions, which are affected by the interaction of childhood trauma and rs6265, and other psychological symptoms that are likely to comorbid with PTSD or cognitive styles that could affect the onset of PTSD in Korean participants.

The purpose of this study was to examine the interaction effect of childhood trauma and BDNF rs6265 on both PTSD symptoms and cortical thickness in Korean participants. In addition, this study was aimed to investigate the relationship of cognitive styles and psychological symptoms with cortical thickness of regions affected by the interaction of childhood trauma and rs6265. We hypothesized that the effect of childhood trauma on both PTSD symptoms and cortical thickness might differ depending on the BDNF rs6265genotype.

## Results

### Descriptive Statistics

The comparisons of demographic, genetic, and psychological characteristics between healthy and PTSD participants are provided in Table 1. The years of education, the childhood trauma questionnaire (CTQ), posttraumatic stress disorder checklist (PCL), posttraumatic growth inventory (PTGI), lifetime events checklist (LEC), hospital anxiety and depression scale (HADS), insomnia severity index (ISI), and ruminative response scale (RRS) scores, and some subscales of the cognitive emotion regulation questionnaire (CERQ) were significantly different between healthy participants and PTSD participants. However, since all variables, including both the CTQ and PCL scores, were normally distributed even for the total participants pool, further analyses with such a pool were executed, based on the assumption of normal distribution to represent the phenomenon in the general population.

**Table 1:** Comparison of the demographic, genetic, and psychological characteristics between participants.

	Total participants (N = 216)	Healthy participants (N = 133)	PTSD participants (N = 83)	*t* *(χ²*)	*p*
	*Mean* ± *SD* or N (%)				
Age (years)	45.67 ± 13.45	46.88 ± 13.54	43.88 ± 13.18	1.552	0.122
**Sex**					
Male	72 (33.33)	40 (30.07)	32 (38.55)	1.653	0.198
Female	144 (66.67)	93 (69.93)	51 (61.45)		
Education (years)	13.16 ± 3.18	13.79 ± 2.91	12.16 ± 3.36	**3.657**	**<0.001**
CTQ	45.87 ± 17.99	42.50 ± 14.92	51.28 ± 21.02	**−3.317**	**0.001**
**BDNF rs6265**					
CC(Val/Val)	67 (31.00)	36 (27.07)	31 (37.35)	2.525	0.112
CT(Met/Val) + TT(Met/Met)	149 (69.00)	97 (72.93)	52 (62.65)		
PCL	27.63 ± 19.59	17.36 ± 13.67	44.10 ± 16.11	**−13.045**	**<0.001**
PTGI	57.80 ± 19.25	61.46 ± 19.85	52.72 ± 17.23	**3.227**	**0.001**
LEC	3.81 ± 2.45	3.19 ± 2.34	4.80 ± 2.32	**−4.935**	**<0.001**
HADS - anxiety	8.50 ± 4.87	6.32 ± 3.50	12.01 ± 4.71	**−9.499**	**<0.001**
HADS - depression	8.71 ± 4.43	6.95 ± 3.70	11.54 ± 4.03	**−8.574**	**<0.001**
ISI	10.96 ± 7.73	7.38 ± 5.87	16.70 ± 6.86	**−10.627**	**<0.001**
AUDIT	3.14 ± 3.69	2.90 ± 3.52	3.52 ± 3.94	−1.164	0.246
RRS	44.58 ± 14.24	39.28 ± 12.11	53.07 ± 13.29	**−7.841**	**<0.001**
CERQ total	108.56 ± 18.72	108.30 ± 17.20	108.98 ± 21.02	−0.259	0.796
putting into perspective	12.73 ± 3.25	13.38 ± 2.91	11.69 ± 3.50	**3.851**	**<0.001**
refocus of planning	14.29 ± 3.30	14.71 ± 3.02	13.61 ± 3.62	**2.309**	**0.022**
positive refocusing	12.04 ± 3.85	12.87 ± 3.43	10.71 ± 4.13	**3.986**	**<0.001**
self-blame	10.98 ± 3.83	10.90 ± 3.27	11.10 ± 4.60	−0.335	0.738
blaming other	10.06 ± 4.06	9.12 ± 3.19	11.57 ± 4.80	**−4.108**	**<0.001**
focus on thought rumination	12.02 ± 3.49	11.38 ± 2.89	13.05 ± 4.09	**−3.244**	**0.001**
acceptance	13.25 ± 3.57	13.62 ± 3.66	12.82 ± 3.39	1.390	0.166
catastrophizing	10.20 ± 4.33	8.36 ± 3.24	13.15 ± 4.25	**−8.781**	**<0.001**
positive reappraisal	12.83 ± 3.85	13.98 ± 3.46	10.99 ± 3.75	**5.984**	**<0.001**

Abbreviations: CTQ, childhood trauma questionnaire; PCL, posttraumatic stress disorder checklist; PTGI, posttraumatic growth inventory; LEC, lifetime events checklist; HADS, hospital anxiety and depression scale; ISI, insomnia severity index; AUDIT, alcohol use disorders identification test; RRS, ruminative response scale; CERQ, cognitive emotion regulation questionnaire.

### Moderated Regression Analyses

To examine the interaction effect of childhood trauma and rs6265 on the PCL score, moderated regression analyses were executed. The CTQ score was set as the independent variable since it represents genetic factors, whereas the rs6265 genotype (Val/Val vs. Val/Met + Met/Met) was set as a moderator. Firstly, the effect of demographic factors such as gender, age, and years of education, and the effect of the experience of traumatic events other than childhood trauma (LEC) were controlled for as covariates to reveal the pure effect of CTQ and rs6265 on PTSD symptoms. In the total participants pool, both the moderation model (*R²* = 0.355, *p* < 0.001) and the moderation effect were significant, given that the *R²* was increased due to the interaction (*ΔR²* = 0.020, *ΔF* = 6.492, *p* = 0.012). Although the rs6265 coefficient was not significant (*B* = 12.375, *p* = 0.060), coefficients of both the CTQ (*B* = 0.480, *p* < 0.001) and the interaction between the CTQ and rs6265 (*B* = −0.326, *p* = 0.012) were significant. Separately, this moderation effect was significant in PTSD participants (*B* = −0.353, *p* = 0.022), but not in healthy participants (*B* = −0.119, *p* = 0.466).

Secondly, another analysis was executed by including the effect of other psychological symptoms such as anxiety, depression, insomnia, and alcohol problems for additional covariates to exclude the effect of comorbid symptoms of PTSD. In the total participants pool, both the moderation model (*R²* = 0.757, *p* < 0.001) and the moderation effect were significant, given that the *R²* was increased due to the interaction (*ΔR²* = 0.005, *ΔF* = 3.954, *p* = 0.048). Although rs6265 coefficient was not significant (*B* = 7.690, *p* = 0.063), coefficients of both the CTQ (*B* = 0.236, *p* < 0.001) and the interaction between the CTQ and rs6265 (*B* = −0.161, *p* = 0.048) were significant. Separately, however, this moderation effect was marginally significant in PTSD participants (*B* = −0.212, *p* = 0.057), but not in healthy participants (*B* = −0.135, *p* = 0.302).

**Figure 1:**
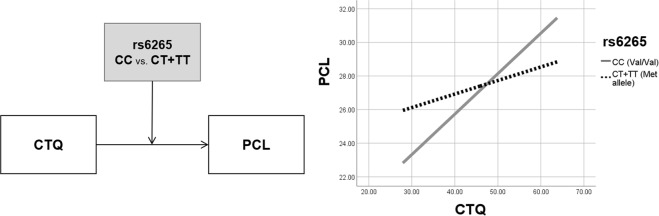
A moderation of the childhood trauma questionnaire (CTQ) on the effect of BDNF rs6265 on the posttraumatic stress disorder checklist (PCL) score.

Results of both moderation analyses are described in Table 2. Although both analyses showed similar results, the second moderation model had much higher *R²* than that of the first moderation model (*R²*_*Model1*_ = 0.335; *R²*_*Model*2_ = 0.757) even with more covariates. Therefore, the second moderation model was accepted and displayed in Fig. 1.

**Table 2:** The moderation effect of the rs6265 polymorphism on the relationship between the childhood trauma questionnaire (CTQ) and the posttraumatic stress disorder checklist (PCL) score while controlling for gender, age, years of education, lifetime events checklist (LEC), hospital anxiety and depression scale (HADS), insomnia severity index (ISI), and alcohol use disorders identification test (AUDIT).

Variables	*B*	*S.E*.	*t*	*R* ^*2*^
**Model 1**				
(Constant)	−30.525	10.331	−2.955**	0.355***
gender	−4.329	2.336	−1.854	
age	−0.203	0.085	−2.408*	
years of education	−1.212	0.359	−3.376**	
LEC	2.353	0.512	−3.376***	
rs6265	12.375	6.553	1.888	
CTQ	0.480	0.109	4.384***	
rs6265 × CTQ	−0.326	0.128	−2.548*	
**rs6265**	**Effect**	**Boot** ***S.E*** *.*	**Boot Confidence Interval**	
CC (Val/Val)	0.480***	0.109	[0.264, 0.695]	
CT & TT (Met allele)	0.154	0.085	[−0.013, 0.321]	
**Model 2**				
(Constant)	−8.607	7.073	−1.217	0.757***
gender	0.949	1.659	0.572	
age	−0.098	0.055	−1.781	
years of education	−0.220	0.231	−0.952	
LEC	0.882	0.331	2.662**	
HADS - anxiety	1.487	0.234	6.361***	
HADS - depression	1.030	0.242	4.257***	
ISI	0.612	0.122	5.028***	
AUDIT	−0.284	0.218	−1.304	
rs6265	7.690	4.107	1.872	
CTQ	0.236	0.070	3.375**	
rs6265 × CTQ	−0.161	0.081	−1.988*	
**rs6265**	**Effect**	**Boot** ***S.E*** *.*	**Boot Confidence Interval**	
CC (Val/Val)	0.236***	0.700	[0.098, 0.374]	
CT & TT (Met allele)	0.076	0.053	[−0.029, 0.180]	

*p < 0.05, **p < 0.01, ***p < 0.001.

As described in Table 2 and Fig. 1, the effect of the CTQ on the PCL was significant in those with the Val/Val (CC) genotype (*B* = 0.236, *p* < 0.001), whereas not in those with the Met allele (CT + TT) (*B* = 0.076, *p* = 0.157). As shown in Fig. 1, people with the Val/Val (CC) genotype showed lower CTQ score than those with the Met allele (CT + TT) when the CTQ score was below 47.500, while people with the Val/Val (CC) genotype showed higher CTQ score than those with the Met allele (CT + TT) when the CTQ score exceeded 47.500.

### Two-way MANCOVA

For further analyses, the total participants pool was used to represent the phenomenon in the general population, instead creating a division between the healthy and the patient groups. Among the total participants pool, the CTQ score was partitioned into two groups, at the point in which the interaction effect of the CTQ and rs6265 was observed, i.e. the score of 47.500. Subsequently, participants were divided into four following groups: (1) Group 1: 36 participants with low CTQ (below 47.500) and rs6562 Val/Val (CC) genotype, (2) Group 2: 99 participants with low CTQ and rs6265 Met allele (CT + TT), (3)Group 3: 31 participants with high CTQ (above 47.500) and rs6265 Val/Val (CC) genotype, and (4) Group 4: 50 participants with low CTQ and rs6265 Met allele (CT + TT).

The two-way MANCOVA was executed on cortical thickness in the total participants pool to examine the effect of low vs. high CTQ, rs6265 Val/Val vs. Met allele, and the interaction effect of the CTQ and rs6265. The demographic factors such as gender, age, and years of education were controlled for as covariates since they could affect cortical thickness, while the use of medication was not considered as a covariate since it was not associated with cortical thickness in previous studies^40,41^. The effect of the experience of traumatic events (LEC) was also controlled for as additional covariate to see the pure effect of the childhood trauma experience on cortical thickness. Considering that several cortical areas were analyzed as dependent variables simultaneously, the Bonferroni correction was used for multiple comparisons (*p*_*adj*_ < 0.00125).

The interaction effect of the CTQ and rs6265 was significant on the thickness of the left fusiform gyrus [*F*(1, 208) = 10.817, *p* = 0.001]. Although it was significantly thinner in participants with the Val/Val (CC) genotype and with high CTQ than in those with low CTQ [*F*(1, 61) = 8.252, *p* = 0.006], it was not significantly different regardless of the CTQ level those with Met allele (CT + TT) [*F*(1, 143) = 1.728, *p* = 0.191]. In addition, left fusiform gyrus thickness was not significantly different regardless of the genotype in people with low CTQ [*F*(1, 129) = 1.001, *p* = 0.319], although it was significantly thicker in participants with high CTQ and with the Met allele (CT + TT) than in those with the Val/Val (CC) genotype [*F*(1, 75) = 11.746, *p* = 0.001].

**Figure 2:**
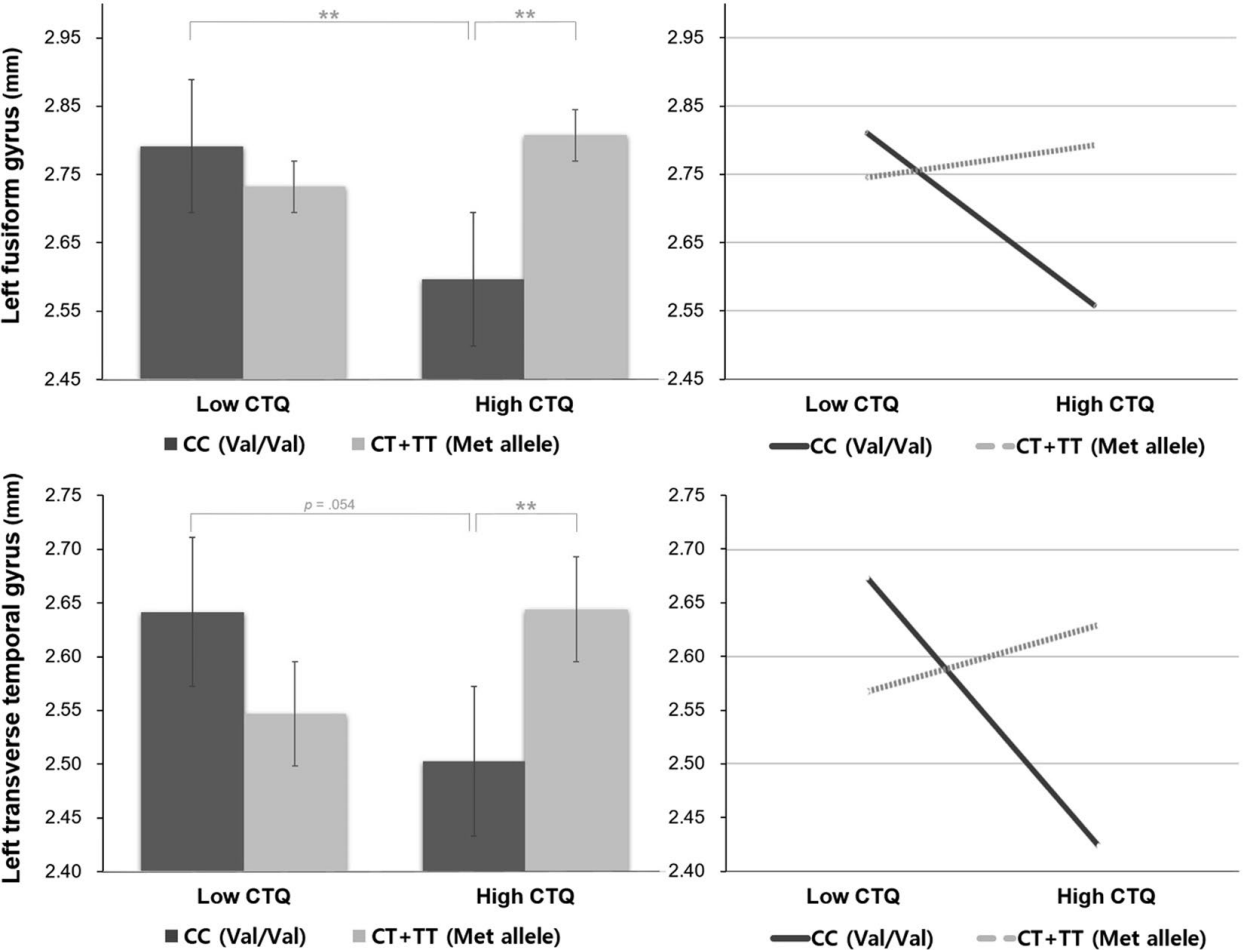
The interaction effect of the childhood trauma questionnaire (CTQ) and rs6265 on both the left fusiform gyrus and the left transverse temporal gyrus.

Additionally, the interaction effect of the CTQ and rs6265 was significant on the thickness of the left transverse temporal gyrus [*F*(1, 208) = 10.604, *p* = 0.001]. In fact, the left transverse temporal gyrus was marginally thinner in participants with the Val/Val (CC) genotype and with high CTQ than in those with low CTQ [*F*(1, 61) = 3.873, *p* = 0.054]. In contrast, it was not significantly different regardless of the CTQ level those with Met allele (CT + TT) [*F*(1, 143) = 1.728, *p* = 0.191]. Furthermore, the thickness of the left fusiform gyrus was not significantly different regardless of the genotype in people with low CTQ [*F*(1, 129) = 3.100, *p* = 0.081], although it was significantly thicker in those with high CTQ and with the Met allele (CT + TT) than in those with the Val/Val (CC) genotype [*F*(1, 75) = 11.746, *p* = 0.001]. These results are presented in Fig. 2.

### Correlation Analysis

**Figure 3:**
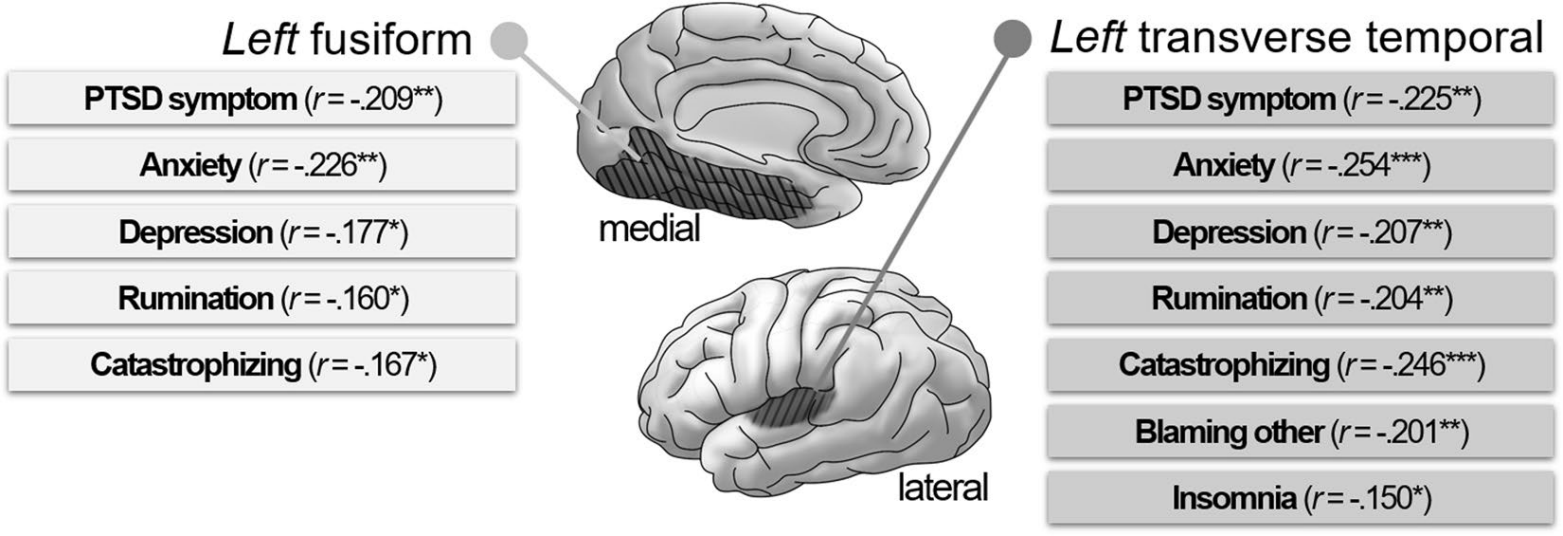
Brain regions affected by the interaction between the childhood trauma questionnaire (CTQ) and rs6265 and the related psychological symptoms.

A partial correlation analysis was executed on both left fusiform and transverse temporal gyri thickness to examine their relationship with psychological symptoms by generating 1000 bootstrapped samples for multiple comparisons^42^. Age was controlled for as a covariate since it was significantly correlated with both the thicknesses of the left fusiform (*r* = −0.217, *p* = 0.001) and transverse temporal (*r* = −0.470, *p* < 0.001) gyri, while gender (*r* = −0.036, *p* = 0.597; *r* = −0.006, *p* = 0.928, respectively) and education years (*r* = −0.057, *p* = 0.405; *r* = −0.002, *p* = 0.978, respectively) were not. As shown in Fig. 3, both the left fusiform and left transverse temporal gyri were significantly associated with the PCL score, both the anxiety and depression scores of the HADS, the RRS score, and the score describing catastrophizing in the CERQ. In addition, left transverse temporal gyrus thickness was significantly correlated with the score describing blaming others in the CERQ and the ISI score.

Further partial correlation analyses were executed on both the thicknesses of the left fusiform and transverse temporal gyri among people with the Val/Val (CC) genotype and the Met allele (CT + TT) for comparison. Again, age was controlled for as a covariate. As shown in Table 3, the correlations between cortical thickness and psychological symptoms differed among genotypes. In fact, the left fusiform gyrus thickness showed a significant negative correlation with PCL, HADS, RRS, and CERQ only in people with the Val/Val (CC) genotype. Similarly, the left transverse temporal gyrus thickness reported significant negative correlations with PCL, HADS, RRS, and catastrophizing in CERQ and a significant positive correlation with positive reappraisal in CERQ and PTGI only in people with the Val/Val (CC) genotype. In contrast, it indicated significant negative correlation with HAD - anxiety, blaming others and catastrophizing in CERQ in people with the Met allele (CT + TT).

**Table 3:** Brain regions affected by the interaction between the childhood trauma questionnaire (CTQ) and rs6265 and the related psychological symptoms between genotypes.

	*r* with left fusiform		*r* with left transverse temporal	
	CC (Val/Val)	CT & TT (Met allele)	CC (Val/Val)	CT & TT (Met allele)
PCL	−0.379**	−0.110	−0.376**	−0.140
PTGI	0.225	−0.052	0.281*	−0.069
HADS - Anxiety	−0.370**	−0.143	−0.355**	−0.19*4
HADS - Depression	−0.273*	−0.113	−0.313*	−0.143
ISI	−0.126	−0.057	−0.223	−0.108
AUDIT	−0.039	−0.014	0.000	−0.105
RRS	−0.372**	−0.060	−0.405**	−0.104
CERQ	−0.265**	0.019	−0.046	−0.073
putting into perspective	−0.008	−0.003	0.144	−0.038
refocus of planning	−0.036	0.144	0.120	0.103
positive refocusing	0.052	−0.102	0.168	−0.076
self-blame	−0.258*	0.041	−0.113	−0.084
blaming other	−0.216	−0.099	−0.154	−0.223**
focus on thought rumination	−0.375**	0.038	−0.179	0.016
acceptance	−0.266*	0.086	−0.074	0.033
catastrophizing	−0.375**	−0.060	−0.348**	−0.183*
positive reappraisal	0.178	0.035	0.350**	−0.031

*p < 0.05, **p < 0.01, ***p < 0.001.

Abbreviations: PCL, posttraumatic stress disorder checklist; PTGI, posttraumatic growth inventory; HADS, hospital anxiety and depression scale; ISI, insomnia severity index; AUDIT, alcohol use disorders identification test; RRS, ruminative response scale; CERQ, cognitive emotion regulation questionnaire.

## Discussion

The current study aimed at examining the interaction effect of childhood trauma and BDNF rs6265 polymorphism on both the PTSD symptoms and cortical thickness among Korean population. People with both the Val/Val genotype of BDNF rs6265 and a CTQ score over 47.500 showed severe PTSD symptoms. Furthermore, the interaction between this gene and childhood trauma also affected both the thickness of the left fusiform and transverse temporal gyri. Thickness, in turn, significantly correlated with symptoms severity, although the relationship differed between genotypes.

Our results revealed that the experience of childhood trauma could predict the severity of PTSD symptoms; however, this relationship was only significant in people with Val/Val (CC) genotype, but not in people with the Met allele (CT + TT) of the BDNF rs6265. In contrast, the BDNF rs6265 genotype did not affect PTSD symptom severity itself. Although previous studies revealed the effect of the rs6265 polymorphism not to be significantly related with PTSD, they did not consider the environmental effect^16,19,23,25,26,43,44^. Therefore, our results validated the hypothesis stating that gene-environment interaction may be the key to understanding the development of PTSD symptoms.

This moderation effect was significant both in the total participants pool (*B* = −0.161, *p* = 0.048), marginally in PTSD participants (*B* = −0.212, *p* = 0.057), but not in healthy participants (*B* = −0.135, *p* = 0.302). These group differences could be a result of dissimilarities in both the pathological characteristics and the genetic predisposition of our groups. For example, PTSD participants reported a higher CTQ mean score with a smaller standard deviation than healthy participants, which could imply the possibility of the floor effect. In addition, the frequency of Met carriers, the protective allele, was slightly higher in healthy participants (73.1%) than in PTSD participants (61.9%).

This interaction effect of childhood trauma and BDNF polymorphism was also found on the cortical thickness of both the left fusiform and left transverse temporal gyri. These regions were in fact thinner in participants with Val/Val genotype and with higher CTQ scores than in those with lower CTQ. Furthermore, both regions resulted thinner in participants with higher CTQ scores and with the Val/Val genotype than in those with the Met allele. Moreover, cortical thickness of both brain areas was negatively associated with scores of PTSD symptoms severity. These results suggest that the gene-environment interaction could influence the thickness of the left temporal regions, which are also related to PTSD symptoms.

Previous studies discovered a significant correlation between the left fusiform gyrus and PTSD^45–47^. The left fusiform gyrus is generally known to be associated with cognition, learning processes^48–50^, negative cognition of PTSD such as dysfunction of memory, dissociative symptoms^51^, and threat appraisal^47^. The current research is in line with these studies since the thickness of the left fusiform gyrus was significantly correlated with PTSD symptoms and other negative cognition (e.g., rumination, blaming others, and catastrophizing). Additionally, the left transverse temporal gyrus, also referred to as Heschl’s gyrus, was described to be linked to PTSD in several studies^52–56^. The left transverse temporal gyrus appears to be involved in assigning a spectral order to pitch information^57^ and language comprehension^58^, which could be related to the low-level perceptual deficits observed in PTSD^59^. Although this study did not evaluate low-level perception, our results highlight the importance of the left transverse temporal gyrus in PTSD.

Other than PTSD symptoms, this study found the relation of left fusiform and transverse gyri with diverse psychological symptoms including anxiety, depression, rumination, insomnia, and some subtypes of cognitive emotional regulation, which is in line with various other studies. The reduced fusiform gyrus thickness is known as a trait marker for vulnerability to depression^60^ and social anxiety^61^. The fusiform gyrus is also considered to be related with the retrieval of memories and rumination^62^. In addition, the thin transverse temporal gyrus was related with depression^63^, and the decreased connectivity in the transverse temporal gyrus was found in high anxiety people^64^, in insomnia patients^65^, and in depressive patients with rumination^66^. Although transverse temporal gyrus is known to be related with exteroceptive sensory perception, a frontoparietal network including this area could be associated with cognitive control as well^67^. Therefore, results of this study support the possibility that these brain regions could act as key roles regarding psychopathology including PTSD and other various symptoms.

Results from this study suggest the BDNF rs6265 Met allele assumes a protective genotype when interacting with childhood trauma; in contrast, the Val/Val genotype may increase the risk of developing PTSD symptoms and cortical thickness. However, previous studies showed mixed results on this matter, as some indicated the Met allele to be risky^8,10,20–22^, while others proposed the Val/Val genotype to be the risky one instead^27,28,33^. A possible reason behind such a difference may be participants’ racial differences^26^. For example, our participants reported higher rs6265 Met allele (66.8%) than did other studies on western participants (approximately 31–40%)^68,69^. This said, the allele frequency found in our study participants was similar to the one reported by other studies (63.64–71.64%) conducted on Korean participants^23,70,71^.

A study with Korean participants revealed an absence of association between the rs6265 and PTSD^23^, while others found the Met allele to act as an allele increasing the risk of developing PTSD^71,72^. However, when considered the interaction of rs6265 and childhood maltreatment, the Val/Val genotype was reported to be associated with a higher anxiety than the Met/Met genotype in Korean females^39^. Furthermore, the participants with the Val/Val genotype showed high harm avoidance traits after recent negative stressors in Korean participants^73^.

Another possible reason behind the mixed results related to the genotype effect may be gene-gene interaction. A few studies suggested in fact that the BDNF genotype may interact with the catechol O-methyltransferase (COMT) and the serotonin transporter gene linked promoter region (5-HTTLPR) to produce differences in resilience^74^ and onset age of depressive disorder^75^, respectively. Further studies investigating such a gene-gene interaction are needed, given that PTSD is known to be related to resilience^76^, depression^77^, and other psychological factors.

Despite significant findings, there are some limitations in this study. Firstly, childhood trauma was assessed retrospectively. Although the CTQ is known to present a stable measurement over time^78^, additional longitudinal studies would be helpful to determine the effect of childhood trauma with accuracy. Furthermore, this study considered the BDNF polymorphism only. Multi gene effects should be explored for better define the gene-environment interaction in future studies, including genome wide association studies. Lastly, neither the severity nor frequency of the traumatic events in PTSD patients were considered as a possible covariate in this study. Further studies considering these effects should be executed in future.

This is one of the first studies to examine the interaction effect of BDNF rs6265 and childhood trauma on both PTSD symptoms and cortical thickness in Korean population. We found that people with both the Val/Val genotype of rs6265 and high CTQ score showed severe PTSD symptoms. Furthermore, this interaction affected the thickness of both the left fusiform and transverse temporal gyri, which significantly correlated with psychological symptoms, although such relationship differed between genotypes. Finally, these results indicate the possibility that the Val/Val genotype may increase the risk of such effect in our Korean population.

## Methods

### Participants

A total of 255 Korean volunteers were initially included in the present study. However, 12 participants were excluded due to missing data from psychological measurements; nine additional participants were omitted from the analyses due to missing MRI and rs6265 data; and 18 participants who showed high denial scores in the childhood trauma questionnaire were also excluded, leaving a final sample of 216 volunteers. While the healthy participants were 133 (61.57%) and recruited through community advertisements, the PTSD patients were 83 (38.43%) and were recruited through notices on the bulletin board in the hospital instead. The PTSD patients were diagnosed based on the Diagnostic and Statistical Manual of Mental Disorders, Fifth Edition (DSM-5) by a psychiatrist, and healthy participants were also assessed using the DSM-5 by a psychiatrist. The PTSD patients were mainly exposed to the following traumatic events: 57 (68.67%) to severe motor vehicle accidents, 7 (8.43%) to defect from North Korea, other 7 (8.43%) to physical or sexual violence, further 7 (8.43%) to relationship issues, 4 (4.82%) to death of a family member, and 1 (1.20%) to fire. The total participants pool was comprised of 72 (33.33%) men and 144 (66.67%) women, with a mean age of 45.67 years (SD = 13.45) and a mean for years of education of 13.16 (SD = 3.18). Specially, healthy participants consisted of 40 (30.07%) men and 93 (39.93%) women, with a mean age of 46.88 years (SD = 13.54). The mean years of education were 13.79 (SD = 2.91). PTSD patients consisted of 32 (38.55%) men and 51 (61.45%) women with a mean age of 43.88 years (SD = 13.18) and a mean for years of education of 12.16 (SD = 3.36). Each participant signed a written form of informed consent, approved by the Institutional Review Board at Inje University Ilsan Paik Hospital prior to the start of the research (IRB no. 2015-07-025), and all measurements and experiments were executed in accordance with guidelines and regulations of the board.

### Psychological measures

Childhood trauma was examined using a Korean validated version of *childhood trauma questionnaire* (CTQ) which consists of five subscales of various childhood traumas, including emotional abuse, physical abuse, sexual abuse, emotional neglect, and physical neglect, and another scale for detecting minimization and denial^79^. The CTQ is comprised of 28 items and it is assessed with a 5-point Likert scale, ranging from 1 (“never true”) to 5 (“very often true”).

To examine the severity of PTSD symptoms, the *posttraumatic stress disorder checklist for DSM-5* (PCL-5) was administered. The PCL is a self-report rating scale for assessing symptoms of PTSD^80^ and was well-validated in Korea^81^. It consists of 20 items and is rated using a 5-point Likert scale, ranging from 1 (“not at all”) to 5 (“extremely”). In addition to PTSD symptoms, The Korean validated version of the *posttraumatic growth inventory* (PTGI) was conducted to measure the degree of reported positive changes experienced after traumatic events^82^. It comprise four subscales, namely changes of self-perception, increase of interpersonal depth, finding new possibilities, and increase of spiritual interest^83^. It consists of 21 items and is rated using a 6-point Likert scale, ranging from 0 (“I did not experience this change as a result of my crisis”) to 5 (“I experienced this change to a very great degree as a result of my crisis”).

To control the exposure to traumatic events other than childhood trauma, the Korean validated version of *Life Events Checklist* (LEC) was used to assess the experience of potentially traumatic events^84^. The LEC comprised of 17 items of PTEs and the responses include experiencing, witnessing, and learning about it. This study analyzed responses of experience since other responses could be confusing to some respondents.

Other psychological symptoms that could possibly comorbid with PTSD were also assessed. The Korean validated version of the *hospital anxiety and depression scale* (HADS) was used to evaluate anxiety and depression symptoms. It consists of 7 items for describing anxiety and 7 items for determining depression^85^, and it is assessed with a 4-point Likert scale, ranging from 0 (no problems) to 3 (maximum distress). Moreover, the Korean version of the *insomnia severity index* (ISI) was used to evaluate the difficulty in falling and staying asleep, problems in waking up too early, the satisfaction with current sleep patterns, the interference with daily functions, the perception of impairment attributed to sleep problems, and the distress caused by the sleep problem^86^. It comprises seven items assessed with a 5-point Likert scale ranging from 0 to 4, with a higher score indicating greater insomnia severity. Additionally, the *Alcohol Use Disorders Identification Test* (AUDIT) was used to assess alcohol consumption, drinking behaviors, and alcohol-related problems before and after experiencing traumatic events. The AUDIT is a 10-item screening tool developed by the World Health Organization, and well-validated in Korea^87^. The AUDIT is assessed with a 5-point Likert scale ranging from 0 (“never”) to 4 (“4 or more times a week”).

Questionnaires that measures cognitive styles which could affect the onset of PTSD were assessed as well. The Korean validated version of the *ruminative response scale* (RRS) was conducted to measure ruminative responses. It is comprised of three subscales, specifically evaluating self-reproach, reflection, and depressive rumination^88^. It consists of 19 items and is rated using a 4-point Likert scale, ranging from 1 (“almost never”) to 4 (“almost always”). Furthermore, the Korean validated version of the *cognitive emotion regulation questionnaire* (CERQ) was applied to measure the specific cognitive emotion regulation strategies used by participants in response to stressful life events. It consists of nine subscales, evaluating putting into perspective, refocus of planning, positive refocusing, self-blame, blaming other, focus on thought rumination, acceptance, catastrophizing, and positive reappraisal^89^. It consists of 36 items and is assessed with a 5-point Likert scale, ranging from 1 (“almost never”) to 5 (“almost always”).

### BDNF genotyping

All participants had their blood sampled to extract their DNA using a NanoDrop® ND-1000UV-Vis Spectrophotometer. Thereafter, their genomic DNA was diluted to a 5 ng/$\mu \ell$ concentration on 96 well PCR plates. TaqMan SNP Genotyping Assays were obtained from Applied Biosystems. Following, the probes were labeled with either the FAM or the VIC dye at the 5′ end and with a minor-groove binder and a non-fluorescent quencher at the 3′ end. PCR was performed in a 5 μl of a mixture containing 2 μl of a DNA sample, 0.125 μl of each TaqMan™ SNP Genotyping Assay (Thermo Fisher Scientific, USA), 2.5 μl of the TaqMan™ Genotyping Master Mix (Thermo Fisher Scientific, USA), and 0.375 μl of distilled water. Amplification and detection were completed with a detection system (QuantStudio 12 K Flex Real-Time PCR System, Thermo Fisher Scientific, USA), using the following profile: 50 °C for 2 min, 95 °C for 10 min followed by 60 cycles of 95 °C for 15 sec, and 60 °C for 1 min. Successively to PCR amplification, allelic discrimination, an endpoint plate read, is performed at the same machines (QuantStudio 12 K Flex Real-Time PCR System). In fact, the QuantStudio 12 K Flex Software calculates the fluorescence measured during the plate read and plots Rn values based on the signals coming from each well. Subsequently, automatic or manual allele calls were performed on the analyzed plates.

Three positive and one negative control samples were present on each plate. We confirmed the clustering image with positive controls, which consisted of. Intra genomic DNA (gDNA) samples of known genotypes are used for positive control.

Sixty-seven participants had the Val/Val (CC) genotype, whereas 149 participants reported the had Met allele (CT + TT) in the rs6265.

### MRI acquisition and processing

A 1.5 T scanner (Magneton Avanto, Siemens, Erlangen, Germany) with restraining foam pads to reduce head motions was used for MRI acquisition. Parameters for MRI images were same as our previous study: a 227 × 384 acquisition matrix, a 210 × 250 field-of-view, 0.9 × 0.7 × 1.2 voxel size, a total of 87,168 voxels, a TE of 3.42 ms, a TR of 1,900 ms, 1.2 mm slice thickness, and a flip angle of 15°^90^.

After visual inspection for artifacts, the surface-based morphometry (SBM) analysis was executed using the Computational Anatomy Toolbox (CAT12, http://www.neuro.uni-jena.de/cat/) toolbox in SPM12 (Wellcome Department of Cognitive Neurology, London, UK). The images were regularized with the International Consortium for Brain Mapping template for East Asian brains and normalized using the Diffeomorphic Anatomical Registration using Exponentiated Lie algebra (DARTEL) algorithm^91^. The images were segmented into gray matter, white matter, and cerebrospinal fluid^92^ using Jacobian-transformed tissue probability maps.

The cortical thickness was estimated with the projection-based thickness method^93^ and extracted using the Desikan-Killany Atlas (DK40), which contains 35 cortical areas in each hemisphere^94^. The cortical thickness value at each vertex was calculated as the closest distance from the gray/white boundary to the gray/CSF boundary^95^.

### Statistical Analysis

Normality was tested using the skewness and kurtosis measures. Skewness over 2.0 and kurtosis over 7.0 are considered to present moderately non-normal distribution^96^. All variables in our results were within the range of normal distribution. After checking for normality, a regression analysis using the SPSS Macro PROCESS for SPSS 2.16.3^97^ was performed to examine the interaction between childhood trauma and rs6265 on the PCL score while controlling for the effect of gender, age, and years of education as covariates. Subsequently, the CTQ score was partitioned into two, at the point in which the moderation effect was first observed. A two-way multivariate analysis of covariance (MANCOVA) was used to find the interaction effect of childhood trauma and rs6265 on cortical thickness, whereas a correlation analysis was conducted to find the relation between cortical thickness and psychological symptoms. All statistical analyses were performed with the SPSS 25 (SPSS, Inc., Chicago, IL, USA). The order and the process of statistical analyses are presented in Table 4. All measurements and all experiment protocols were approved by the Inje University Ilsan Paik Hospital Institutional Review Board (IRB no. 2015-07-025) and were executed in accordance with guidelines and regulations of the board.

**Table 4:** the process of analyses and reasons for statistical methods.

Process	Analyses	Reasons for Analyses
1	Normality Testing	to examine the interaction normal distribution for further analyses
2	Moderated Regression Analysis	to examine the interaction effect of childhood trauma and rs6265 on PTSD symptoms and discover the score where the interaction happens
3	Multivariate Analysis of Covariance	to examine the interaction effect of childhood trauma and rs6265 on cortical thickness based on the score of the interaction
4	Correlation Analysis	to examine the relationship between psychological symptoms and cortical thickness of regions affected by the interaction of childhood trauma and rs6265

## Data Availability

The data that support the findings of this study are available from the corresponding author, S.-H.L., upon reasonable request.
